# X-Ray Spectrometry of Copper: New Results on an Old Subject

**DOI:** 10.6028/jres.109.006

**Published:** 2004-02-01

**Authors:** M. Deutsch, E. Förster, G. Hölzer, J. Härtwig, K. Hämäläinen, C.-C. Kao, S. Huotari, R. Diamant

**Affiliations:** Physics Dept., Bar-Ilan University, Ramat-Gann 52900, Israel; Institute for Optics and Quantum Electronics, Friedrich-Schiller-University Jena, D-07743 Jena, Germany; ESRF, B.P. 220, F-38043 Grenoble Cedex, France; Division of X-Ray Physics, Department of Physical Sciences, P.O.B. 64, FIN-0014 University of Helsinki, Finland; NSLS, Brookhaven National Laboratory, Upton, NY 11973, U.S.A; Division of X-Ray Physics, Department of Physical Sciences, P.O.B. 64, FIN-0014 University of Helsinki, Finland, (Present address: ESRF, BP 220, F-38043 Grenoble Cedex, France); Physics Dept., Bar-Ilan University, Ramat-Gann 52900, Israel

**Keywords:** copper, hypersatellites, multi-electronic transitions, satellites, x-ray spectra

## Abstract

We review recent, and some less recent, measurements of several emission spectra of copper. The results are discussed with special emphasis on elucidating the structure of the K*α*_1,2_ and K*β*_1,3_ diagram lines and their underlying transitions. These lines are found to contain ≈30 % contribution from 3*d* spectator hole transitions. Other multielectronic transitions, the 2*p* spectator hole (satellites) and 1*s* spectator hole (hypersatellites) transitions were also measured. They are discussed paying special attention to the evolution of the lineshapes and intensities from the excitation threshold to saturation. Trends in the measured quantities depending on the spectator hole’s shell and subshell are also discussed.

This review is dedicated to the memory of two pioneers, Teijo Åberg and Dick Deslattes, who led, each in his own way, generations of researchers to a deeper and more comprehensive understanding of the Physics of X Rays.

## 1. Introduction

A wavelength conveniently close to the repeat distance of many crystals, reasonably low absorption in low- and medium-*Z* elements, and favourable physical and chemical properties facilitating the construction of x-ray tubes conspired to make the Cu K*α* x rays the most widely used radiation in diffraction and scattering experiments. These wide-employed applications, and the intriguing asymmetry of the Cu K*α* and K*β* lineshapes [1,2], along with those of all 3*d* transition elements [3–8], led, in turn, to a century of extensive spectrometric studies of the Cu K*α* and K*β* spectra. In spite of these extensive studied, recent studies reveal that surprises still lurk under the skewed K*α*_1,2_ and overlapping K*β*_1,3_ lines, and the related multi-electronic satellite (S) and hypersatellite (HS) spectra.

The asymmetric lineshape of the copper emission lines were attributed in the past to a number of different processes: Kondo-like interaction of the conduction electrons with the core-holes [1,2,9], final-state interactions between the core holes and the incomplete 3*d* shell [6], 2*p*/3*d* shell electrostatic exchange interaction [7] and, most importantly, shake-up and shake-off of electrons from the 3*l* shells [8,10]. The last process, in particular, received in the past strong experimental support [10–12]. Our measurements of both K*α*_1,2_ and K*β*_1,3_ diagram spectra to be described below [13–15], carried out in conjunction with relativistic multiconfigurational Dirac-Fock (RMCDF) *ab initio* calculations, also supports this interpretation, and provide accurate numerical estimate of the magnitude of the contribution from these effects to the lineshape. Moreover, we have carried out recently excitation-energy-dependent measurements of the spectra near the excitation energy threshold for these shake-off effects. Our results lend further support to the assignment of the asymmetry of the Cu diagram lines to 3*d* spectator-hole transitions (in the following, underlining denotes hole states).

To support a detailed and accurate shape analysis, high resolution measurements of the spectra are required. This, in turn, requires a detailed study of the various factors affecting the resolution of the measuring system, and their optimization for a given measurement. Thus, in preparation for the Cu K*α*_1,2_ and K*β*_1,3_ measurements, we have carried out a careful study and characterization of our measuring system [16]. This allowed to minimize the spectral distortions due to finite resolution. Also, our use of a calibrated silicon analyzer allowed an uncertainty on the order of 10^−6^ (K*β*) or better (K*α*) in the absolute energy scale. The use of the same well characterized experimental setup under well defined resolution conditions for both the K*α* and K*β* spectra provided higher confidence in the conclusions drawn from the data. We have also carried out *ab initio* relativistic Dirac-Fock calculations to help determine the structure underlying the lines. These assumed that the spectral lineshapes can be accounted for by satellites resulting from 3*l* spectator hole transitions in addition to the nominal single electron diagram transitions. We have found a specific set of transitions which accounts extremely well for the K*α*, and to a somewhat lesser extent, also for the K*β* lineshapes [13–15].

The theoretical understanding of the diagram lines, even when considering only the dominant single-electron-transition components, becomes more complicated when the near-threshold region is considered. In the “isothermal” region, high above threshold, the excitation and de-excitation processes of inner shell electrons have been extensively studied [17], and can be well described theoretically by the prevailing Sudden- or Frozen-Core approximation, where the ejected electron is removed immediately, the atom’s shell structure is kept frozen in its ground state configuration, and the electrons treated as independent, non-interacting and uncorrelated entities [18]. The excitation and de-excitation processes are treated in this regime as two independent and consecutive processes. By contrast, in the “adiabatic” regime [19], near the excitation threshold, effects like inter- and intra-shell electronic correlations, atomic shell relaxation and the changing interaction between the slow-moving ejected electron and the relaxing atom, become progressively more important, even when *single*-electron transitions are involved [18,20,21]. Near threshold, the excitation process, and the de-excitation by photon (x rays) or electron (Auger) ejection can no longer be considered to be independent processes; they become increasingly simultaneous, mutually interacting, and merge into a single complex process. In this regime the Sudden and Frozen-core approximations are no longer valid in principle. Thus, the study of the near threshold region is very important for elucidating electronic correlations and the interplay between excitation, relaxation and emission in atoms.

To study these effects, we have used tunable synchrotron radiation to excite the diagram lines at near-threshold energies and follow the evolution of the spectral shape and intensity from below threshold to saturation [22,23]. These measurements, reported below for the first time, indicate a threshold for the asymmetry of the lines at ≈10 eV above the K edge. Below this threshold the Cu K*α* and K*β* lines are found to have symmetric Lorentzian shapes. The asymmetry was found to grow with excitation energy and to saturate ≈100 eV above threshold. These results strongly support the assignment of the asymmetry to 3*d* spectator transitions, the threshold of which is expected to occur for Cu at ≈8 eV to 10 eV above the K edge, according to the *Z*+1 approximation [24].

Spectator-hole transitions, like the 3*d* spectator-hole transitions contributing to the diagram lines, involving more than a single electron or vacancy, go beyond the simple single electron, frozen atom description of the excitation and emission processes. They require taking into account correlations between electrons in the same, or in different shells [18]. Such multielectronic effects can best be studied in satellite (2*p*-spectator transitions, denoted K*α*_3,4_, originating in 1*s*2*p →* 2*p*^2^ transitions) and hypersatellite (1*s*-spectator transitions, denoted K*^h^α*_1,2_, originating in 1*s*^2^ *→* 1*s*2*p* transitions) spectra, which, on the one hand, originate in transitions involving two vacancies in their initial and final states, and, on the other hand, are removed from, and thus not masked by the strong diagram lines, as are the 3*d* spectator lines straddling the K*α* and K*β* diagram transition lines. The study of satellite and hypersatellite spectra, particularly near threshold, was hampered until very recently by the lack of suitable excitation sources which are tunable, narrow-band, and intense enough to allow studying these weak transitions. Pioneered by Deslattes and coworkers [25], and further developed by others, synchrotron-based beamlines equipped with efficient, high resolution fluorescence spectrometers have become available over the last few years [26,27]. Such facilities allow detailed studies of this kind, as well as inelastic scattering experiments, to be carried out [22,25,28]. We will demonstrate here the richness and sophistication achievable in studies using this powerful technique by discussing briefly two of our recent studies: the CuK*α*_3,4_ satellites [22,23], and the CuK*^h^α*_1,2_ hypersatellites [29,30].

We have studied the variations in the Cu K*α*_3,4_ emission spectrum (ranging from 8060 eV to 8100 eV) as a function of the photoexcitation energy from threshold at ≈10000 eV up to the saturation of the satellites’ intensity at 11 200 eV. The results confirm the pure shake-off nature of the spectrum, as predicted by theory. They also reveal two distinct regimes in the spectral evolution. In the first, up to ≈50 eV above threshold, the spectral shape as well as the intensity undergo a rapid and complicated variation with excitation energy. In the second regime, from ≈50 eV to ≈1000 eV above threshold, no variation of the spectral shape is observed, and only the overall intensity increases monotonically to saturation at the range’s upper end. The saturated spectral shape, though not the fast-varying one near threshold, is found to be in good agreement with *ab initio* RMCDF calculations, which allows to identify the various spectral features with specific transitions. The Thomas Model [31,32], developed specifically for the cross-section evolution in the “adiabatic” regime near threshold employing time-dependent perturbation theory, though successful in the case of valence electrons in low-*Z* atoms [33], does not agree well with our experimental results, indicating a strong need for more detailed theoretical work in this almost unexplored regime.

Hypersatellites (HS), are of particular interest for a number of reasons. First, since the initial state involves two electrons in the same shell, the spectra should allow studying *intra*-shell correlations. Also, since even for medium-*Z* atoms the K shell is already rather strongly relativistic the HS allow studying the effects of relativity on electronic correlations in atoms. Moreover, the K*^h^α*_1_ HS line originates in the ^1^S_0_ *→* ^3^P_1_ spin flip transition, which is dipole-forbidden in the pure *LS* coupling scheme and fully allowed only in the *jj* coupling scheme [24,34]. Thus, the intensity ratio *R* = *I*(K*^h^α*_1_)/*I*(K*^h^α*_2_) is strongly dependent on the degree of intermediacy of the coupling [35], rendering it the most sensitive (and almost only) method for quantitatively studying the coupling variation across the periodic table from the *LS* coupling limit at low *Z* to the *jj* coupling limit at high *Z*. K*^h^α*_1,2_ HS spectra are also unique in allowing to study the Breit interaction, the most elusive and least studied of all atomic interactions. Because of a near cancellation of the Coulomb terms, the contribution of the Breit interaction to the splitting of the K*^h^α*_1,2_ HS lines, and to their shift from the diagram lines, can reach as high as 20 % at high *Z*, rather than the small 1 % contribution in the case of diagram lines [36,17]. Finally, HS are the “diagram” transitions of, and thus allow studying *hollow atoms* [37], i.e., atoms where a whole inner shell is empty, while the outer shells are occupied [38]. The formation mechanism of such atoms and their properties are of fundamental interest to basic atomic physics [37,40]. They are also of great importance for studies of atoms very far from equilibrium and of ultra-fast dynamics in atoms, with possible wide-ranging applications in physics, chemistry, biology and materials science [41], as well as a possible way towards the realization of hard-x-ray lasers [41,42].

Using the same technique employed for the Cu satellites, we have measured the first clean, resolved Cu K*^h^α*_1,2_ hypersatellite spectrum [29,30]. The line splitting and shift from the diagram line agree well with our, and previous, RMCDF calculations. Nevertheless, these calculation slightly overestimate the measured intensity ratio *R*. A typical shake-off behaviour with intensity increasing smoothly from threshold is found, in agreement with shake theory predictions and previous satellite measurements. The Thomas Model does not agree well with the measured intensity evolution, as also found for satellites. The evolution of the spectrum’s intensity from threshold shows an unexpectedly large saturation range, ≈60 % of the threshold energy. Taken together with the results, mentioned above, obtained for the 3*d* and 2*p* spectator transitions, a distinct trend emerges in the length of the saturation range with orbital quantum number *l*. To the best of our knowledge, such a trend has not hitherto been addressed by theory.

In the following we discuss the experimental and calculational methods, the results obtained for the diagram lines, and, briefly, also the satellite and hyper-satellite results.

## 2. Experiment and Calculations

### 2.1 Single and Double Crystal Laboratory-Based Spectrometry

The laboratory-based diagram line measurements were carried out using a conventional sealed x-ray tube source, operated at 40 kV and 30 mA. For accurate wavelength measurements a single-crystal spectrometer was employed, while for the lineshape measurements a double-crystal spectrometer was used. Both spectrometers were optimized using the detailed theoretical and experimental study of Härtwig et al. [16].

The single crystal spectrometer allowed absolute angle measurements with a mean total angle dividing error of 0.12″ arc-sec and an angular step size of 0.06″ [43]. Using the WASO9 silicon crystal [44], previously calibrated at the German institute of standards, PTB, this spectrometer allowed, in principle, wavelength measurements to an accuracy of ∆*λ*/*λ* = 3 × 10^−7^ [44,45]. For the CuK*α* spectrum the symmetrical (444) reflection was used and for the CuK*β* spectrum- the asymmetrical (553) reflection. The incident beam was defined by a 390 mm long collimator, with horizontal slits 0.42 mm high and 0.03 mm (CuK*α*) and 0.10 mm (CuK*β*) wide. Under these conditions the measured intensity distribution is well approximated by a convolution of the true spectral distribution and the (approximated) instrumental function. The true distribution was obtained in this case by deconvolution of the measured spectrum.

The double crystal spectrometer is a commercial DTS spectrometer [46]. Since the zero angle of this device could not be determined accurately, the energy scale was fixed by defining the angular position of the maximum of the CuK*α*_1_ peak to be *λ*_0_ = 0.154 059 292 nm [44]. For the double crystal spectrometer it was possible to make the instrumental function very narrow, so that no correction for finite resolution was necessary, and only a correction for absorption was applied to the data [16]. Here symmetric Si(333) reflections were used in both crystals for both spectra. A 560 mm long collimator was employed with a horizontal slit width of 1.0 mm (focus side) and 10 mm (sample side) for both spectra, and vertical slit heights of 0.4 mm (CuK*α*) and 0.8 mm (CuK*β*).

Further details of the experimental setup of the diagram line measurements are given in Refs. [13,15].

### 2.2 Photoexcited Synchrotron-Based Spectrometry

**Fig. 1:**
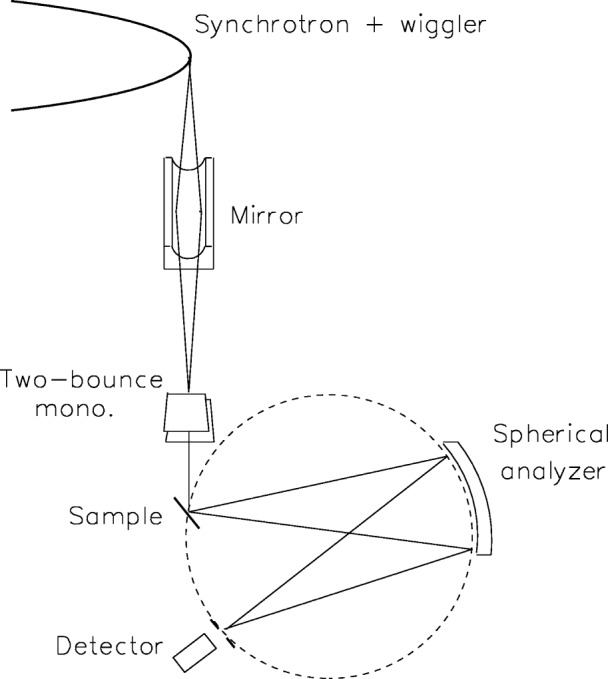
Schematic view of the synchrotron-based setup for measuring near-threshold photoexcited emission spectra. The incident exciting beam is monochromatized by a double-bounced silicon crystal monochromator. The emitted radiation is recorder by a Johann-type spectrometer using a spherically-bent analyzer in near-back-reflection geometry.

The measurements in the threshold region of the diagram lines, and all the satellite and hypersatellite spectra, were carried out at beamlines X21 and X25 at NSLS, Brookhaven National Laboratory. The experimental setup is shown schematically in Fig. 1. In the X25 beamline, for example, the source is a hybrid 27-pole wiggler. The radiation from the source is focussed by a toroidal mirror, followed by a 2-bounce Si (220) or a Si (111) monochromator. This provides an incident energy resolution of 2 eV to 6 eV, and a flux of ≈10^12^ photons/s in a spot size of ≤ 0.7 mm^2^ at the sample. The incident intensity is monitored by an ionization chamber. The fluorescence radiation from the sample is measured by a Johann type spectrometer, having a Rowland circle of 1 m diameter on a horizontal plane, and a spherically bent 3 in diameter Si analyzer. For the diagram K*α*_1,2_ (at ≈8050 eV), the satellite K*α*_3,4_ (at ≈8080 eV), and the hypersatellite K*^h^α*_1,2_ (at ≈8330 eV) spectra the Si(444) reflection was used. For the diagram K*β*_1,3_ (at ≈8900 eV) the Si(553) reflection was used. For the energies studied the analyzers have high Bragg angles, ranging from 70° to 80°, which, in conjunction with the small focus of the exciting radiation, provide for a resolution of 2.8 eV or lower for these spectra. A liquid nitrogen cooled intrinsic Germanium detector was used to measure the analyzer-focussed radiation. Further details, especially on the beamline and spectrometer, measurement procedures and data treatment are given in [22,23,26].

### 2.3 *Ab Initio* Transition Calculations

The calculations employed in the data interpretation in all studies were done *ab initio*, using the relativistic multiconfigurational Dirac-Fock (RMCDF) package GRASP [47], with supplementary code written in-house. Following previous work [21,48] the initial and final state wavefunctions were generated in separate, independent runs. The energies of the individual transitions are then obtain by subtracting the appropriate level energies, as calculated in the initial- and in the final-state runs. This procedure was found to yield accurate energies for the CuK*α*_3,4_ satellites even for excitation energies within 50 eV of the threshold [22].

The calculation of relative transition probabilities within each multiplet requires the wavefunctions of the initial and final states to be orthogonal. Since the initial and final states are generated here in separate runs, this condition is not fulfilled. Thus, as in previous studies [13,22,49], configuration interaction calculations were carried out to obtain the various transition probabilities using once the initial state orbitals and again those of the final state. All the significant transition probabilities in the two sets agreed with each other to within ± 10 %. We used, therefore, the line strengths calculated from the initial state wavefunctions.

### 2.4 Phenomenological and Theoretical Spectral Fits

To obtain an accurate analytical, albeit phenomenological, representation of the measured data the diagram lines were phenomenologically fitted by a sum of Lorentzians, using non-linear least-squares methods. The number of Lorentzians in the sum was increased until a good *χ* ^2^ value, which did not decrease further upon increasing the number of Lorentzians, was obtained. Such a representation is particularly important for the diagram K*α* and K*β* spectra, which are used in diffraction and scattering experiments.

For interpreting the structure of the spectra, the “stick diagram” multiplets obtained in the calculations above were also fitted to the measured data. Here, each transition line was replaced by a single Lorentzian, the integrated area of which was equal to the relative intensity of the corresponding “stick” within the multiplet. Usually, all Lorentzians representing lines within a given multiplet were assigned a common width, although in some cases (particularly for the diagram spectra interpretation) two independent widths were used: one for transitions associated with the *α*_1_ or *β*_1_ lines, and the other for transitions associated with the *α*_2_ or *β*_3_ lines. A spectrum was typically fitted by a sum of several multiplets, where for each multiplet a single intensity parameter was refined in the fit, representing its relative intensity contribution to the given spectrum. Further details are given in [13,15,25].

## 3. Results and Discussion

### 3.1 The K*α*1,2 and K*β*_1,3_ “Diagram” Spectra

#### 3.1.1 Analytical Representation of the Spectra

**Fig. 2:**
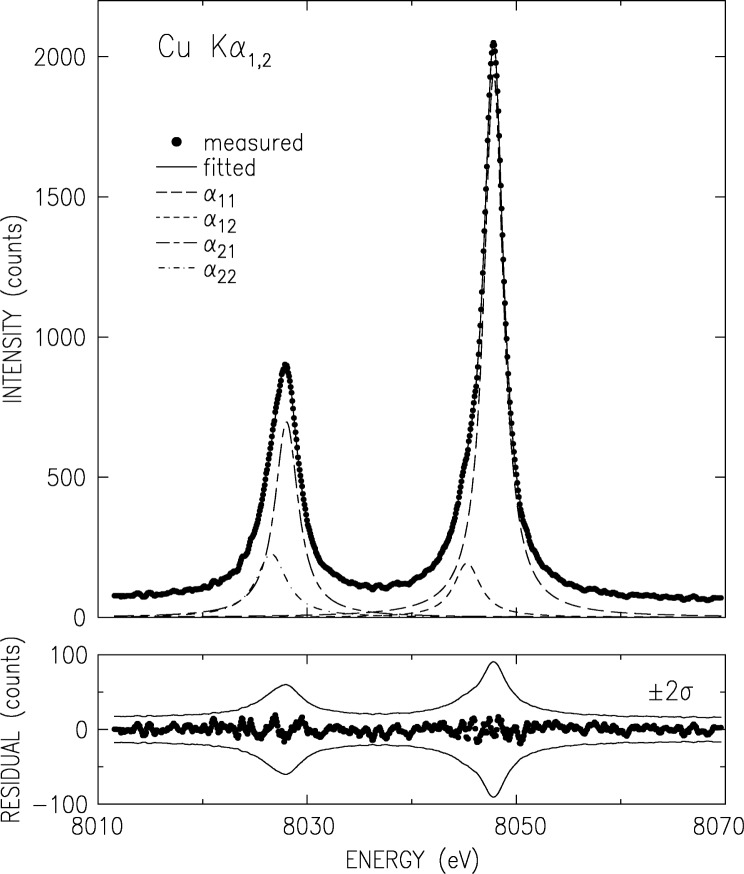
Double-crystal spectrometer measured and smoothed CuK*α*_3,4_ spectrum (points) and fitted Lorentzians. The fit parameters are given in Table 2. The lower panel shows the fit residuals. The thin lines denote the ±2σ values of the data, where σ is the standard deviation.

**Fig. 3:**
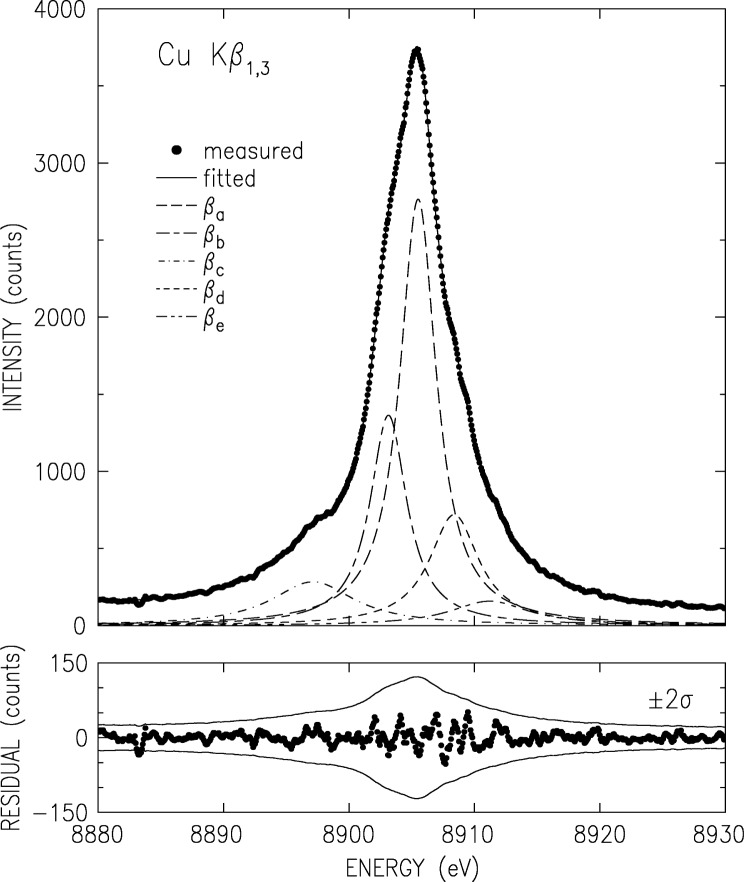
Same as Fig. 2 but for CuK*β*_1,3_.

The K*α* and K*β*, measured using the double crystal spectrometer as discussed above, are shown in Figs. 2 and 3. The basic parameters derived from the measured spectra [15] are summarized in Table 1. A full analysis of this data is given in Ref. [15]. Here we mention only the ±3 × 10^−7^ (K*α*_1,2_) and ±4 × 10^−6^ (K*β*_1,3_) relative accuracy of the absolute wavelengths, the highest to date for these lines. The removal of the finite resolution effects by optimizing the spectrometer to a level of a negligible resolution width (in the case the double crystal spectrometer) or by the deconvolution of the data by a well-determined resolution function (in the case of the single-crystal spectrometer) yields accurate intrinsic lineshapes, which allow carrying out a detailed theoretical analysis, as discussed below.

For analytic representation purposes, the K*α* spectrum was fitted by four Lorentzian, as shown in Fig. 2. The Lorentzians are defined by their position *E_ij_*, width *W_ij_* and relative intensity *I_ij_*, where *i* = 1,2 denotes K*α*_1,2_, respectively, and *j* is the index within each line. The fit parameter values are given in Table 2. The fit residuals (lower panel in Fig. 2) are small and non-systematically scattered around zero. Moreover, they are within the ±2*σ* limits of the data, where *σ* denotes the standard deviation of the data points due to counting statistics. This residual distribution indicates an excellent fit, also demonstrated by the weighted *R*-factor of 0.7 % obtained in the fit.

The double-crystal-spectrometer-measured K*β* spectrum is shown in Fig. 3. As noted earlier [50] four underlying lines are discernible. These were denoted as the two diagram lines *β*_1_ and *β*_3_ and two satellites; *β*′ on the low energy side, and *β″* on the high energy side. A fifth line was proposed by Bremer and Sørum [12], but no determination of its position, intensity and width was carried out. Hayasi [51] also observed this feature and determined its energy without a detailed fit. Bremer and S∅rum also observe a very sharp dip just off the peak, on the low energy side, where only a smooth shoulder was observed in earlier measurements. This was assigned to the spin-orbit splitting of the two diagram lines. No such dip was observed in any of the other published line shapes, nor in our measurements. Finally, although the position of the K*β*′ feature as measured by Salem et al. [52] is in good agreement with theory, its intensity disagrees with both the exchange interaction [52,53] and plasmon oscillation [54] theories.

To address these issues, we have carried out a series of fits with four and five Lorentzians [13]. We found that indeed the use of five Lorentzians yields a significant improvement in the fit residuals and *R* factors over the four-Lorentzian one [13]. The parameters defining the five-Lorentzian representation of the lineshape, as obtained from the fit, are listed in Table 2. Although the weighted *R*-factor of this fit, 1 %, is somewhat higher than that of the K*α* spectrum, the fit is still very good, and, as can be seen in the lower panel of Fig. 3, the residuals are within the ±2*σ* limits of the measured points. The association of the Lorentzians *β*_a_ to *β*_e_ with the phenomenologically determined features *β*_1_, *β*_3_,*β*′, and *β″* of the spectrum is discussed in Ref. [13].

Further details of the various quantities derived directly from the raw K*α* and K*β* spectra, the phenomenological resolution of these spectra into sums of Lorentzians, and their comparison with previous measurements are given in Refs. [13,15,16].

#### 3.1.2 Comparison With *Ab Initio* Theory

**Fig. 4:**
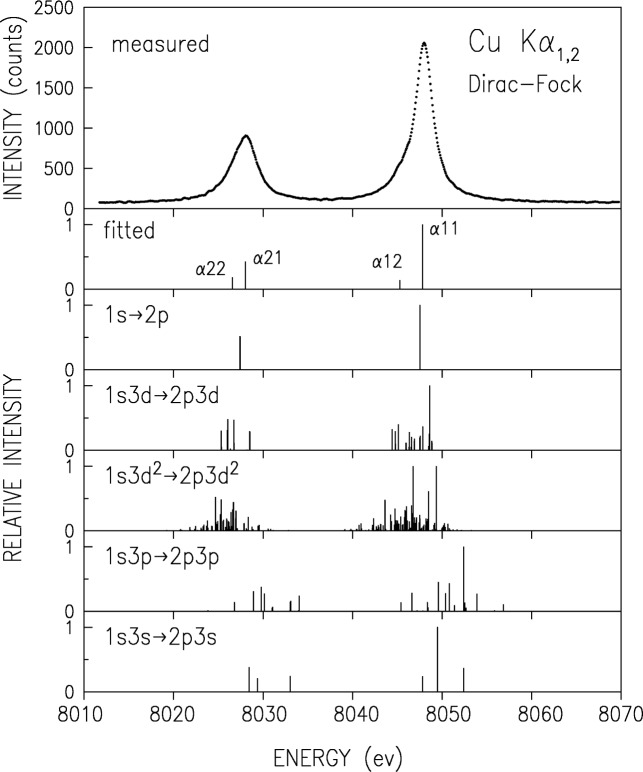
Measured, lorentzian-fitted, and RMCDF-calculated transitions for the K*α* spectrum, including the diagram transition, the 3*s*, 3*p*, and 3*d* one-hole-spectator, and the likeliest 3*d*^2^ two-hole-spectator transitions. Calculated intensities in each multiplet are relative to the strongest line of that multiplet.

**Fig. 5:**
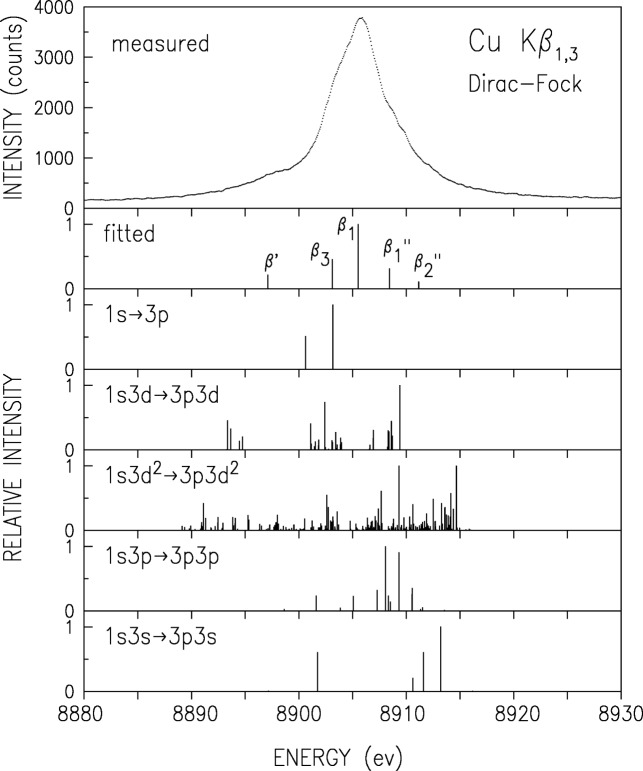
Same as Fig. 4 but for CuK*α*_1,3_.

To elucidate the structure underlaying the diagram lines, we carried out RMCDF calculations for transitions originating in several different initial states. In additional to the diagram transitions 1*s* → 2*p* we considered also the two-hole transitions 1*s*3*l* → 2*p*3*l* where *l* = *s*, *p*, *d*, and the most probable three-hole transition 1*s*3*d*^2^ → 2*p*3*d*^2^. The resulting “stick” diagrams of these multiplets are given in Figs. 4 and 5 for the K*α* and K*β* spectra, respectively, along with the measured spectra and the individual Lorentzians’ positions in the phenomenological fits described above. One can observe in these plots that all multiplets considered are well-aligned with the spectra (within the ≤ 2 eV computational accuracy [13]), and thus can, in principle contribute to the measured spectrum. Since the lifetime widths of the transitions are larger than the interline distances, each of these multiplets will result in a smooth lineshape of overlapping contributions and it is not possible to identify by eye the individual contributions of the various multiplets. Thus, only general conclusions can be drawn from eye inspection. For example, the strongest lines of the 1*s*3*p* → 2*p*3*p* multiplet are in a region where the measured intensity is small, and hence the contribution of this multiplet to the line shape can not be large. A visual inspection of Fig. 5 also reveals that the 3*d* spectator hole multiplet has strong lines in the vicinity of the *β*′, *β″*_1_, and *β″*_2_ features, as pointed out by LaVilla [11].

**Fig. 6:**
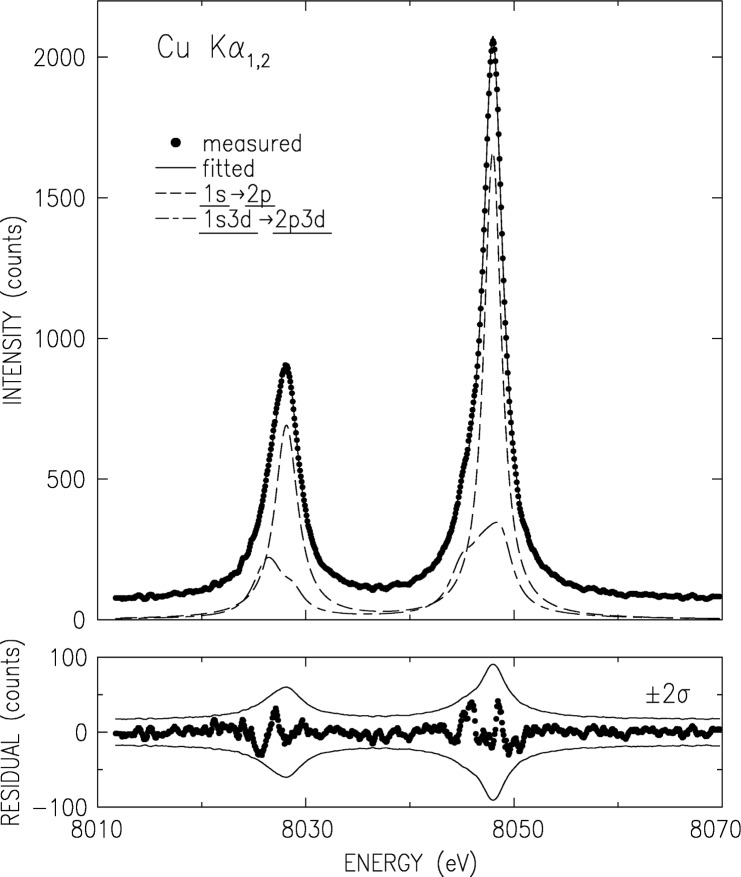
The fit of the best combination of RMCDF-calculated multiplets to the measured CuK*α*_1,2_ spectrum. The fit residuals are shown in the lower panel.

**Fig. 7:**
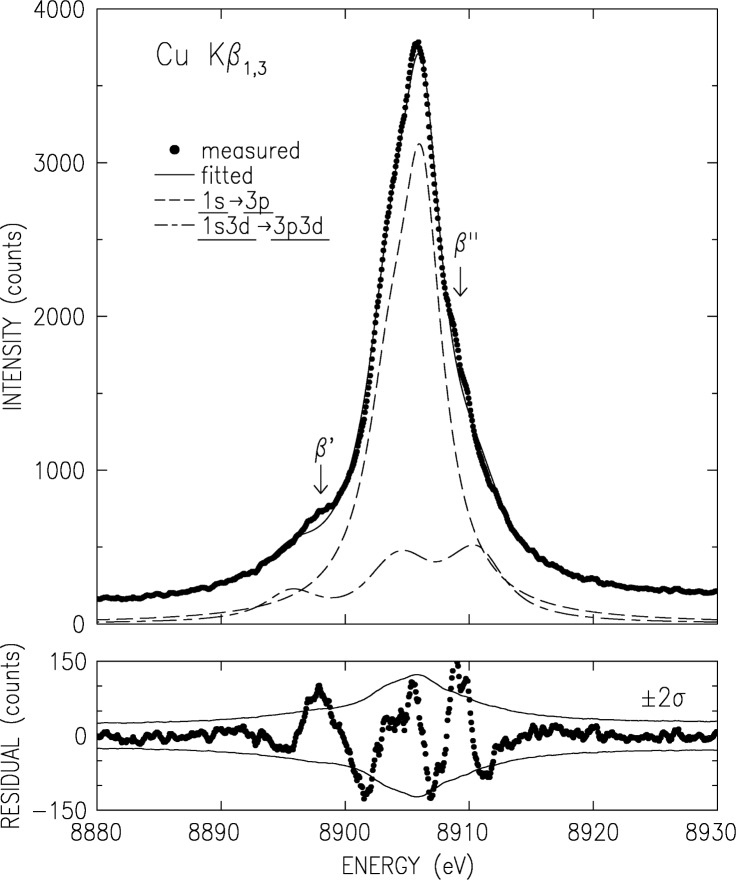
Same as Fig. 6 but for CuK*β*_1,3_. spectrum. Note the deviations in the fit near the marked satellites’ positions.

To obtain more quantitative conclusions, we have carried out a series of computer fits, where each “stick” in a given calculated multiplet was represented by a Lorentzian, whose integrated intensity (area under the curve) is equal to the height of the corresponding “stick”. The widths of all Lorentzians within a multiplet were constrained to be equal. A series of fits with different combinations of the diagram lines with one or more multihole multiplets were carried out, refining the width, overall intensity and position of each multiplet. The details of the fits are given in Tables III and VII in Ref. [13] and will not be reproduced here. The *R* factors and Goodness-of-Fit indicators presented there lead clearly to the conclusion that the inclusion of the 3d spectator transition, 1*s*3*d* → (2*p*, 3*p*)3*d*, in the fit along with the diagram ones, 1*s* → (2*p*, 3*p*), is mandatory for obtaining a good fit for both of the measured K*α* and K*β* spectra. No indication of contributions from the 3*s* spectator transitions was found. This is not surprising in view of the fast depopulation of the 3*s* vacancies by Coster-Kronig transitions. Similarly, no indication of contributions from the 3*p* spectator transitions were found for K*β*, though a very small contribution, ∼0.5 % of the total intensity, to the K*α* spectrum may be possible. The best fit, that including the diagram and the 3*d* spectator transitions is shown in Figs. 6 and 7 for the two spectra, along with the residuals. Note that all residuals of the K*α* are within ±2*σ*, and are randomly distributed. For the K*β*, some deviations of the residuals from this limits occur, due to the *β*′ (≈8897 eV) and the *β″* (≈8908 eV) satellites [11]. However, the small residuals and the good weighted *R* factors, 3 % for K*α* and 5.3 % for K*β*, clearly support the conclusion that the only appreciable contribution to the lines besides the diagram transitions is from the 3*d* spectator hole transition, in agreement with earlier results [10,7] based on non-relativistic HF calculations.

Finally, we note that the best fit, shown in Figs. 6 and 7, yields a rather high contribution of the 3*d* spectator transition to the total intensity of the spectrum: 26 % to 30 % in both spectra. This demonstrates clearly that multielectronic effects play a more important role than hitherto assumed in determining the spectral shape of even the strong diagram lines. Similar or even stronger influence can be expected in the weaker atomic spectra.

#### 3.1.3 The Near-Edge Evolution of the Spectra

The interpretation above for the structure underlying the K*α* and K*β* spectra entails different thresholds for the symmetric (diagram) and the asymmetric (3*d* spectator) contributions to the lineshapes. The symmetric part is due to a single-electron transition, originating in a single 1*s* vacancy. Thus, its threshold is at the binding energy of a single 1*s* electron in a neutral Cu atom. This is the conventional K edge, at 8979 eV [55]. The asymmetric part, due to the 3*d* spectator transition 1*s*3*d* → (2*p*, 3*p*)3*d* originates in a double 1*s*3*d* vacancy. Using the *Z*+1 approximation [24] and the ≈8 eV binding energy of the 3*d* electrons in Zn [55], the threshold for the 3*d* spectator transition, the KM edge, should be about 8 eV to 10 eV above the K edge. An immediate implication of this conclusion is that below this KM edge, but above the K edge, the spectator transition is not excited and the Cu K*α* and K*β* lineshapes should be symmetric and well-represented by a single Lorentzian for each transition. Two points need be noted. Since the 3*d* electron binding energy rises fast with *Z* from Cu up, the actual KM threshold may occur at an energy higher than 8 eV above the K edge. Moreover, as shake-off may dominate the creation of the initial 1*s*3*d* vacancy state, and since the cross-section for a shake-off process rises gradually from zero at threshold, the contribution of the 3*d* spectator transition also rises gradually from threshold. Immediately above threshold the contributions from the 3*d* spectator transitions are still small. Thus, the asymmetric features of the lineshape may not become discernibly until a somewhat higher excitation energy is reached.

**Fig. 8:**
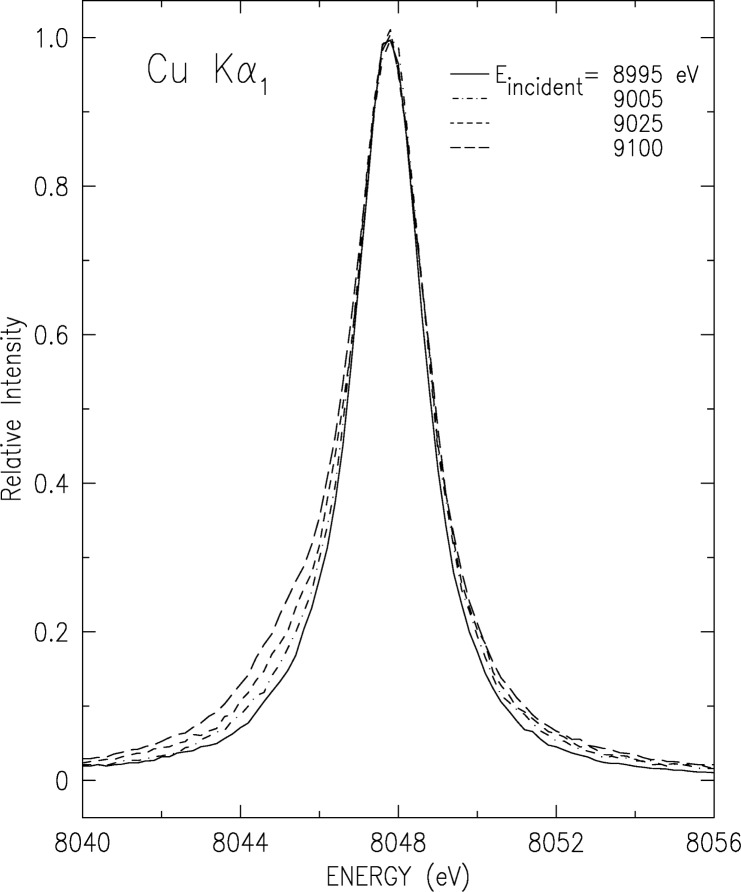
The evolution of the CuK*α*_1_ line with photoexciting energy near threshold from an almost symmetric single-Lorentzian lineshape (solid line) to an increasingly asymmetric lineshape. The asymmetry results from the openning and subsequent growth of the 3*d* spectator hole transition channel starting from 10 eV to 15 eV above the K edge [*E* (K edge) = 8979 eV].

**Fig. 9:**
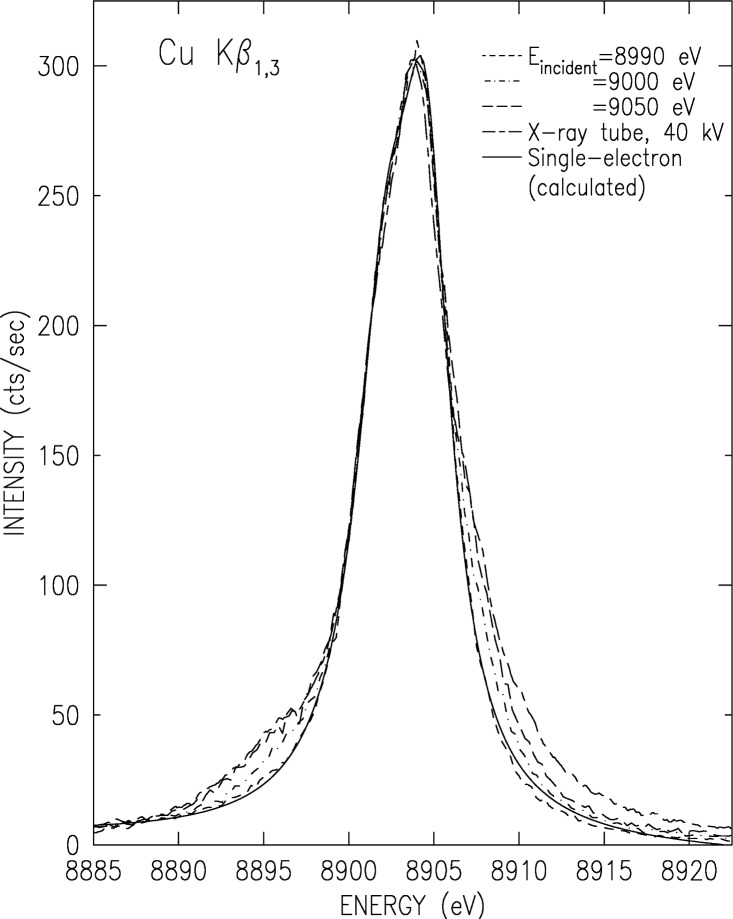
Same as Fig. 8, but for CuK*β*_1,3_. Here the lineshape below and at threshold of the 3*d* spectator hole transitions is that of two overlapping Lorentzians (solid line) as expected of the two overlapping diagram transitions K*β*_1_ and K*β*_3_.

**Fig. 10:**
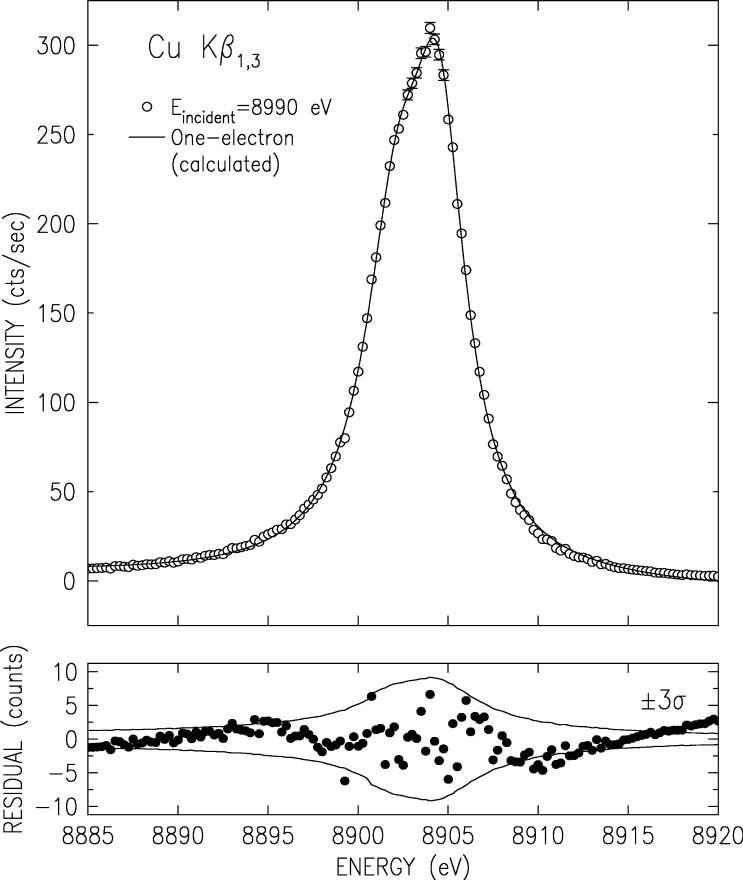
Fit of the measured CuK*β*_1,3_ spectrum above the K edge but (roughly) at the 3*d* spectator-hole-transitions’ threshold. Note the good fit to the two Lorentzians representing the pure diagram transitions, without any indications of asymmetric contributions to the lineshape. The lower panel shows the fit residuals.

We have measured the evolution of CuK*α* and K*β* spectra as the photoexciting energy varies from the K edge and up, using beamline X21 at NSLS, Brookhaven National Laboratory, and the experimental setup described above. The results for the K*α*_1_ line are shown in Fig. 8, and those for the overlapping K*β*_1,3_ complex in Fig. 9. The additional structure, which causes the lineshapes’ deviation from simple Lorentzians and eventually makes them asymmetric, can be clearly seen to increase from an excitation energy of E_excitation_ = 8890 eV and up. As observed in Fig. 10, particularly in the very small fit residuals in the lower panel of the figure, the K*β*_1,3_ spectrum excited at *E*_excitation_ = 8890 eV can indeed be excellently fitted by two Lorentzians only, representing the two one-electron K*β*_1_ and K*β*_3_ diagram transitions: 1*s* → 3*p*_1/2,3/2_. Careful examination of the growth of the 3*d* spectator features in both spectra with increasing excitation energy reveals that the multielectronic features grow monotonically, and reach their maximal intensity about 100 eV to 150 eV above the KM threshold. This range is in good agreement with recent measurements of a 250 eV to 300 eV growth range of the GeK*β′′′* satellites, which originates in similar 3*l* (*l* = *s*, *p*, *d*) spectator transitions [56].

Further details of our measurements of the near-edge evolution of the spectral features of the K*α* and K*β* spectra, with special emphasis on the evolution of features associated with spectator vacancy transitions, were published elsewhere [57].

#### 3.1.4 Monolithic Silicon Spectrometer Measurements

We finish this brief review of our measurements of the CuK*α* and K*β* spectra by a short discussion of a novel, though by now not so new, monolithic crystal device allowing *absolute* wavelength determinations at 10^−6^ level at every laboratory without the use of a previously calibrated crystal.

**Fig. 11:**
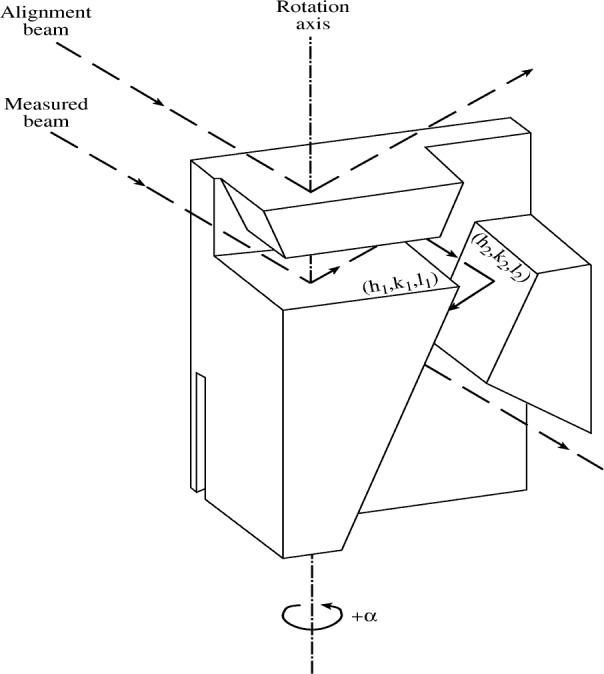
The monolithic double crystal spectrometer (MDCS). *α* is the angle of rotation and (*h*_1_ *k*_1_ *l*_1_) and (*h*_2_ *k*_2_ *l*_2_) are the two participating Bragg planes. The beam trajectory used for aligning the rotation axis normal to the (*h*_1_ *k*_1_ *l*_1_) plane, and the trajectory of the measured beam are also shown.

The Monolithic Double Crystal Spectrometer (MDCS) [58–60] is shown in Fig. 11. It is an x-ray optical element cut from a single block of a perfect crystal. Two sets of Bragg planes of the monolith play the roles of the two separate crystals of a conventional double-crystal spectrometer (DCS) [61]. The requirement of satisfying two Bragg conditions simultaneously determines the transmitted wavelength, given the angle between the two reflecting planes. Energy scanning is done by rotating the MDCS by an angle *α* about the normal to the first set of Bragg planes. This leaves the angle of incidence (Bragg angle) on the first set of planes unchanged, but varies the angle of incidence on the second set, and consequently also the wavelength transmitted by the MDCS. This method of wavelength scanning was pioneered in conventional double crystal spectrometry by Deslattes [62] and employed since in numerous other studies [63]. Nevertheless, the first detailed theoretical study of the MDCS was published only recently [60].

**Fig. 12:**
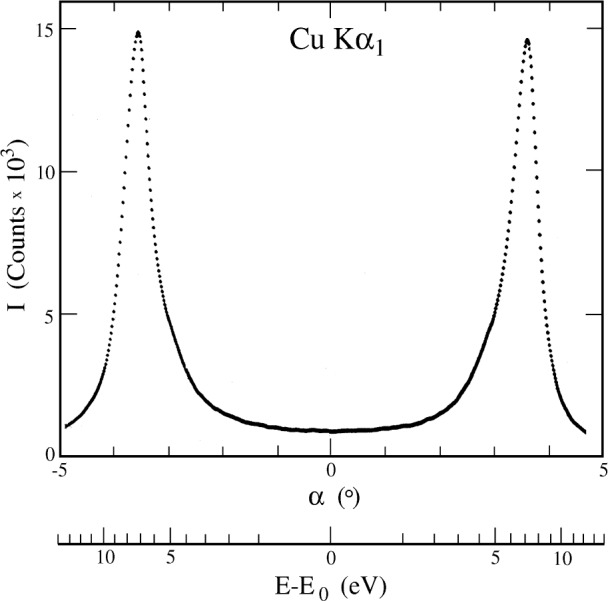
The Cu K*α*_1_ line as measured with negative and positive rotations *α* of the MDCS in Fig. 11, cut from a perfect silicon crystal using the (111) and the $\left(\bar{2}06\right)$ planes.

For an x ray to be transmitted through the MDCS at a given angle of rotation *α*, its wavelength has to be [60]

$$\lambda (\alpha )/d_{1}=2\sin\beta_{0}\cos\alpha /{\left[{\left(\sqrt{\left(h_{2}^{2}+k_{2}^{2}+l_{2}^{2})/(h_{1}^{2}+k_{1}^{2}+l_{1}^{2}\right)}-\cos\beta_{0}\right)}^{2}+\sin^{2}\beta_{0}\cos^{2}\alpha \right]}^{1/2}$$ (1)

here *d*_1_ is the spacing of the first of the two sets of planes, (*h*_1_, *k*_1_, *l*_1_) and (*h*_2_, *k*_2_, *l*_2_), and *β*_0_ is the dihedral angle between these two planes. Two important points should be noted. First, the wavelength transmitted is directly related in Eq. (1) to the lattice constant $a_{0}=d_{1}\sqrt{h_{1}^{2}+k_{1}^{2}+l_{1}^{2}}$ Since particular commercial brands of single crystal silicon boules are now produced routinely to part-per-million tolerances for the lattice constant, cutting an MDCS from such a boule allows *absolute* wavelength determination of an x-ray line to 10^−6^ with moderate effort and simple equipment available in standard x-ray laboratories [58] without having to resort to calibrated crystal slabs obtained from (few and often busy) standards laboratories. For example, the particular MDCS used to measure the spectrum in Fig. 12 below yields *λ* (CuK*α*_1_)/*a*_0_= 0.283 665 49(24), i.e., an uncertainty of 0.85 × 10^−6^ [58]. Second, in a conventional DCS the transmitted wavelength *λ* ∝ sin *α* and, consequently, the wavelength change upon rotation of the second crystal is linear in the angle of rotation: Δ*λ* ∝ Δ*α*. For the MDCS, however, the transmitted wavelength according to Eq. (1) is approximately *λ* ∝ cos *α* so that for small rotation angles the change in the wavelength is quadratic in the angle of rotation: Δ*λ* ∝ (Δ*α*)^2^. A significantly larger rotation is required, therefore, for the MDCS than for the DCS to obtain the same wavelength change. This provides considerably higher angular resolution and dispersion for the MDCS. The CuK*α*_1_ line, measured using a silicon MDCS with (111) and $\left(\bar{2}06\right)$ planes, is shown in Fig. 12. The quadratic dependence of the energy on the rotation angle is clearly observed. This quadratic dependence of Δ*λ* on *α* for the MDCS has several other implications. First, the wavelength transmitted depends on the absolute value of *α* only, not on its sign. Thus, the same spectrum is measured twice; once for the negative and once for the positive senses of rotation. This symmetry relative to *α* = 0 allows to determine accurately the zero point of the rotation from the measured data, for example, as the mid-point between the same peak measured for +*α* and −*α* rotations. This double measurement also allows the identification of features appearing in one spectrum only as spurious, so that it can be excluded from the consequent analysis. These advantages come at a cost of a limited scan range, and the existence of a high limit for the measurable wavelength, *λ*_0_, corresponding to the zero angle, *α* = 0, position of the MDCS:

$$\lambda_{0}/d_{1}=2\sin\beta_{0}/{\left[{\left(\sqrt{\left(h_{2}^{2}+k_{2}^{2}+l_{2}^{2})/(h_{1}^{2}+k_{1}^{2}+l_{1}^{2}\right)}-\cos\beta_{0}\right)}^{2}+\sin^{2}\beta_{0}\right]}^{1/2}.$$ (2)

This limit has to be kept in mind in choosing the Bragg planes to be used in an MDCS designed for scanning a given energy range. To obtain the highest dispersion and resolution, the planes have to be chosen such that the longest wavelength of the range is as close as possible to, but still smaller than, *λ*_0_. Further details can be found in Refs. [58,59], which also discuss in detail the sources and magnitudes of the experimental errors of the MDCS. A detailed theoretical analysis of the MDCS is given in Ref. [60].

### 3.2 The Kα_3,4_ Satellite Spectrum

Using the same technique employed for the near-threshold region of the diagram K*α* and K*β* spectra, we have measured the spectral evolution of the CuK*α*_3,4_ satellite complex from threshold up. The measurements were carried out at beamline X25, NSLS, Brookhaven National laboratory. The full results of that study have been published recently [23,23], and will be reviewed here only briefly.

A two regime behaviour was found in the evolution: in the first ≈50 eV above threshold, a fast variation of both the shape and intensity of the spectrum is found. Above that, only the intensity keeps growing monotonically with excitation energy, without any changes occurring in the spectral shape, until the intensity saturates at ≈1 keV above the threshold. We now discuss each regime separately.

**Fig. 13:**
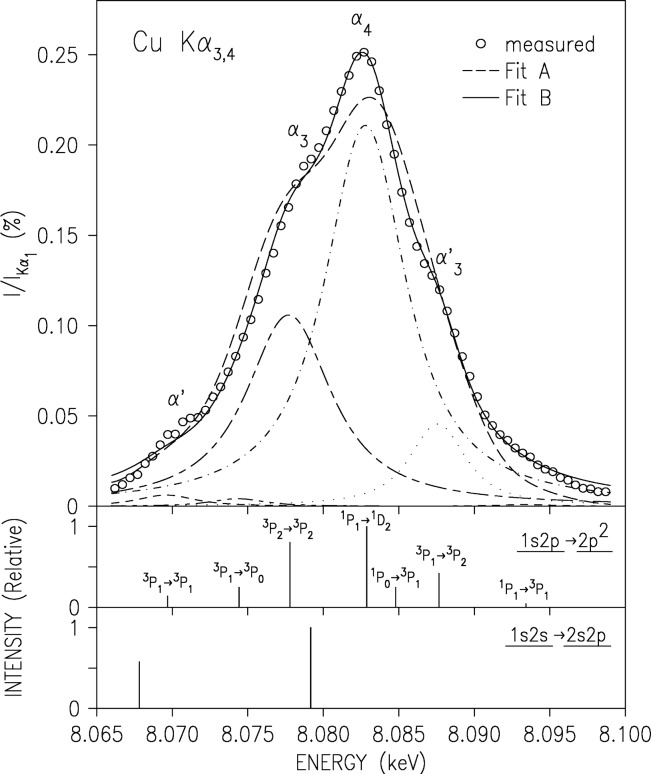
The measured CuK*α*_3,4_ satellite spectrum (points), with its fit by the ab-initio RMCDF calculations. The fits are based on the 1*s*2*p →* 2*p*^2^ transitions shown as a “stick diagram” in the lower panels. Note the good agreement. The lines in the second panel are marked by the LS notation of their major constituents. The calculated 1*s*2*s →* 2*s*2*p* transitions are shown in the third panel. The fits reveal that these transitions do not contribute to the lineshape to any detectable level.

In Fig. 13 we plot the background-subtracted spectrum measured in the high-energy regime. We also plot the RMCDF *ab initio* calculated transition lines corresponding to the 2*p* spectator transitions 1*s*2*p →* 2*p*^2^, which were suggested already in 1927 [64,65] to be the originators of the K*α*_3,4_ spectrum. The overall alignment of the calculated lines with the measured spectrum and, in particular, its various features is good. The four main features, *α′*, *α*_3_, *α*_4_, and *α′*_3_ can clearly be identified with the ^3^P_1_ *→* ^3^P_1_, ^3^P_2_ *→* ^3^P_2_, ^1^P1 *→* ^1^D_2_, and ^3^P_1_ *→* ^3^P_2_ transitions. We have also measured the energy threshold for exciting each of the four main features and found them to be 10017 eV, 10011 eV, 10027 eV and 10015 eV, for the *α′*, *α*_3_, *α*_4_, and *α′*_3_ lines, respectively [23]. The *ab initio* “stick” diagram multiplet of the 2*p* spectator transitions was fitted to the measured spectrum, using a Lorentzian to represent each transition (i.e., each “stick”). Two fits are shown in Fig. 13. Fit A employed a single width common to all the multiplet lines, and only this width, a single intensity scale factor, and a small computational shift (≈1 eV to 2 eV) between the energy scales of the measurement and the calculation were refined in the fit. Fit B allowed the widths and relative intensities of the individual “stick” to vary independently, and only the individual line positions within the multiplet were held fixed. The agreement of even the more restricted fit, Fit A, with the measured spectrum is good, indicating that no other multiplets contribute to the lineshape. Indeed, attempts to include contributions from the calculated 2*s* spectator transitions 1*s*2*s →* 2*s*2*p* invariably reduced their intensity to zero [22]. This is in line with the ≈5 fold lower shake probability calculated for a 2*s* electron, as compared to a 2*p* one, to accompany a 1*s* vacancy production [66]. The very strong Coster-Kronig transition 1*s*2*s →* 1*s*2*p*3*l*, which depopulates the 2*s* spectator state very fast, further reduces any possible 2*s* spectator contributions to the spectrum [13,67]. Finally, we wish to point out that the fact that the as-calculated *ab initio* multiplet (Fit A) requires adjustments, albeit small, of the individual lines’ integrated intensities (via their widths) to achieve a near-perfect fit (Fit B) indicates that while our calculation captures the essentials of the spectrum, further effects, such as slightly less-than-full relaxation and/or final- and initial-state correlations, may need to be included in the *ab initio* calculations to achieve a good agreement with the measurements.

**Fig. 14:**
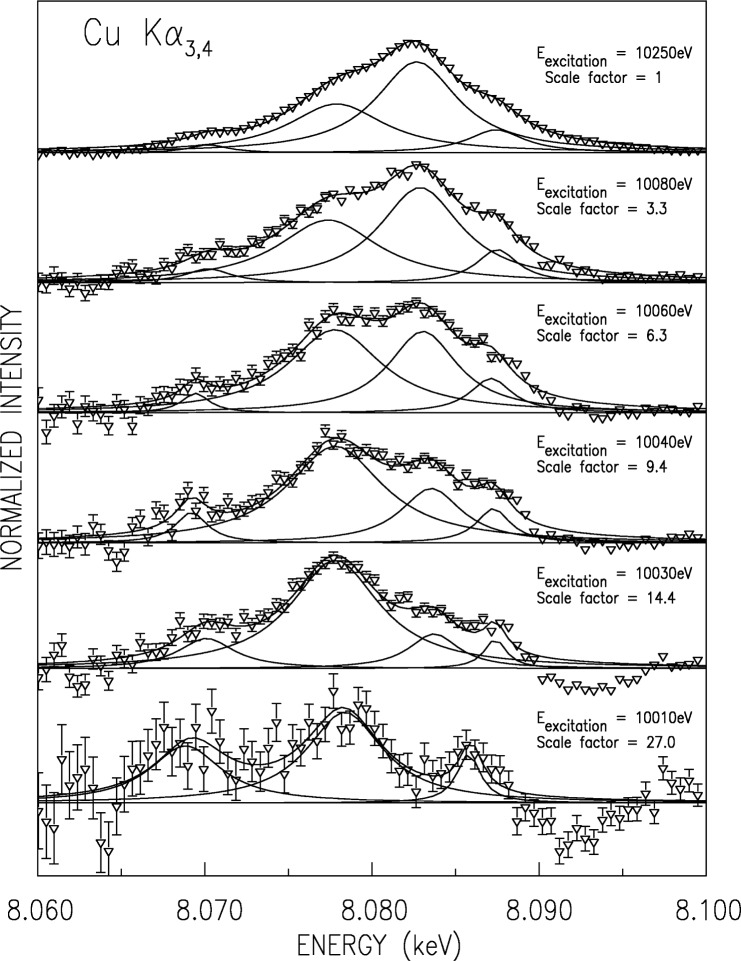
The measured CuK*α*_3,4_ spectra in the near threshold adiabatic region, for several excitation energies, each normalized to its maximum by the scale factor listed. Fits to a sum of four Lorentzians, and the individual Lorentzians, are shown in solid lines. Note the roughly constant widths and positions of the lines, and the increase of the *α*_4_ line from zero below threshold to domination at ~10 080 eV.

Within 50 eV of the threshold, the shape of the spectrum varies rapidly. Several of the spectra measured in this regime are shown in Fig. 14. Each spectrum is normalized to its maximal intensity, so that the weakest spectrum, at *E*_excitation_ = 10010 eV, is about 30-fold less intense than the strongest one, at *E*_excitation_ = 10250 eV. This intensity decrease, and the high background due to the K*α*_1_ tail, effectively prevented meaningful satellite spectrum extraction at lower *E*_excitation_ values. Note that for these reasons, and the finite energy spread in the exciting beam, remnants of some of the emission lines are still observed at the lowest *E*_excitation_ spectrum, measured at the nominal threshold of 10 010 eV. We have used a 4-Lorentzian phenomenological fit to allow following the variation of each underlying line, since the weaker lines could not be refined with any confidence level. For obvious reasons we have not attempted to reproduce the complicated variation of the lines’ intensities with incident energy, observed in this figure, by *ab initio* calculation. This would require including inter-shell electronic correlations and other near-threshold effects in the calculations. We hope that the availability of our measurements in this “adiabatic” regime will spur theoretical developments which will eventually allow *ab initio* calculations to be carried out even in this highly complex regime.

Further details, in particular of the variation of the satellites’ intensity with exciting energy from threshold to saturation, are discussed in Refs. [22,23]. Similar measurements for satellites of Ge were published recently by Sternemann et al. [56]

### 3.3 The CuK*^h^α*_1,2_ Hypersatellite Spectrum

The CuK*^h^α*_1,2_ hypersatellite (HS) measurements were carried out by beamline X25 with the same setup as those of the CuK*α*_3,4_ satellites discussed above. A full discussion is given in Refs. [29,30], and only a short summary is presented here.

**Fig. 15:**
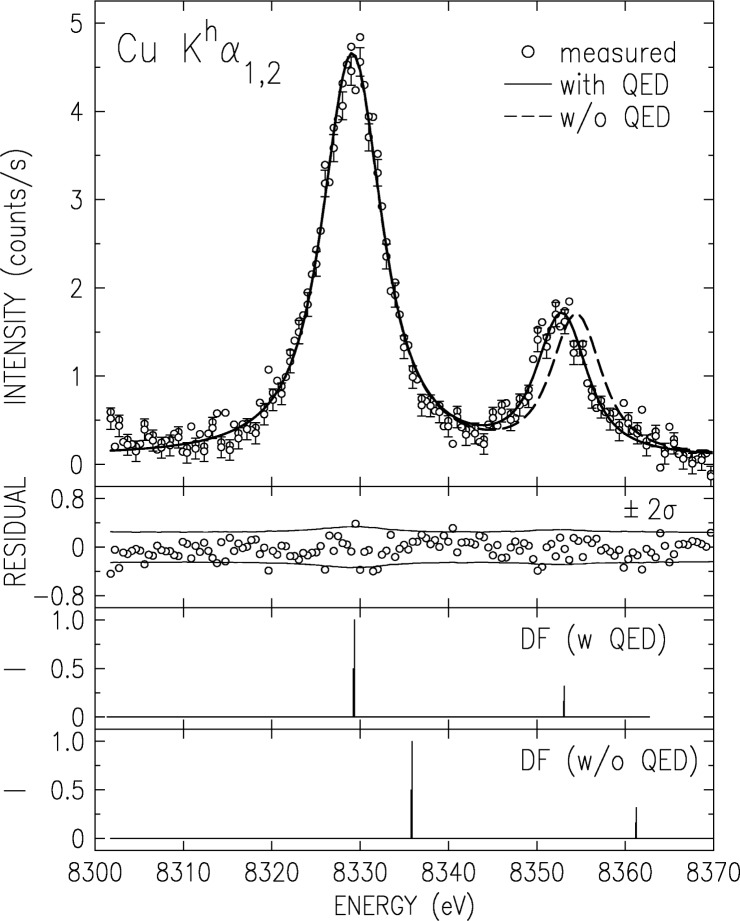
The measured CuK*^h^α*_1,2_ hypersatellite spectrum (points) with its fit (solid line) by the RMCDF calculated “stick diagram” in the third panel. The fit residuals are shown in the second panel. Note the good fit when QED corrections are included in the calculations. The spectrum calculated without these corrections (fourth panel) is upshifted relative to the measured spectrum. It can not be fitted to the measured spectrum even when downshifted, due to its too large line splitting (dashed line). This demonstrates the large contribution of QED effects to the hypersatellite spectra.

The HS spectrum measured at *E*_excitation_ = 20 keV is shown in Fig. 15. The most outstanding feature of the spectrum is the inverted K*α*_1_/K*α*_2_ intensity ratio, ≈0.29, as compared to the well-known value of ≈2 observed for the corresponding diagram lines. This results from the fact that in the LS coupling, prevailing for low-*Z* atoms, the K*^h^α*_1_ transition is spin-flip forbidden. It is fully allowed only in the *jj* coupling, prevailing for high-*Z* atoms. This makes this ratio the most sensitive measure for the intermediacy of the coupling at a given *Z* [68–70]. The value we measured for Cu, 0.29(2), is slightly overestimated by the best RMCDF calculations [68], including ours [30], albeit by no more than two standard deviations.

We have carried out *ab initio* RMCDF calculations of the HS spectrum, both including and excluding QED corrections. The closed 3*d* shell of Cu and the empty K shell of the initial state results in a sparse calculated “stick” diagram, shown in the third and fourth panels of Fig. 15. Representing each line in this diagram by a Voigt function with a fixed Gaussian resolution width of 2.8 eV, we fitted the calculated spectra to the measured one. The resultant fit for the calculation which includes QED is shown in a solid line in Fig. 15. The residuals are shown in the second panel of the figure. They are almost all within the ±2*σ* limits of the measurements. Thus, within the measurements’ statistics, the intrinsic lineshape is well described by a single Lorentzian, indicating no contamination by higher-order multivacancy transitions, such as the 3*d* spectator transitions, which were shown above to contribute 26 % to 30 % to the total intensity of the corresponding K*α*_1,2_ diagram lines. This is not surprising in view of the very low probability for creating the two-K-hole initial state of the HS transition, 10^−4^ relative to that of creating a single K hole, and the fact that the spectator states require one additional hole, i.e., a three-vacancy initial state. The vanishingly low probability of exciting such states is responsible, then, for the high purity of the photoexcited HS spectra. The fit to the calculation which neglects the QED corrections is shown in dashed line in the first panel of Fig. 15. As can be seen, the incorrect splitting does not allow to obtain a good fit to the measured spectrum even when the calculated spectrum is shifted to lower energies by the large amount of ≈7 eV required. This demonstrates the exceptionally large contribution of QED effects to the HS, and hence the importance of the HS spectra for studying these effects [68–70]. Comparing the measured spectrum with more detailed calculations, which include the various components of the QED corrections one at a time should elucidate the relative importance of each of these contributions. Such calculations are however outside the scope of our (mostly experimental) study.

**Fig. 16:**
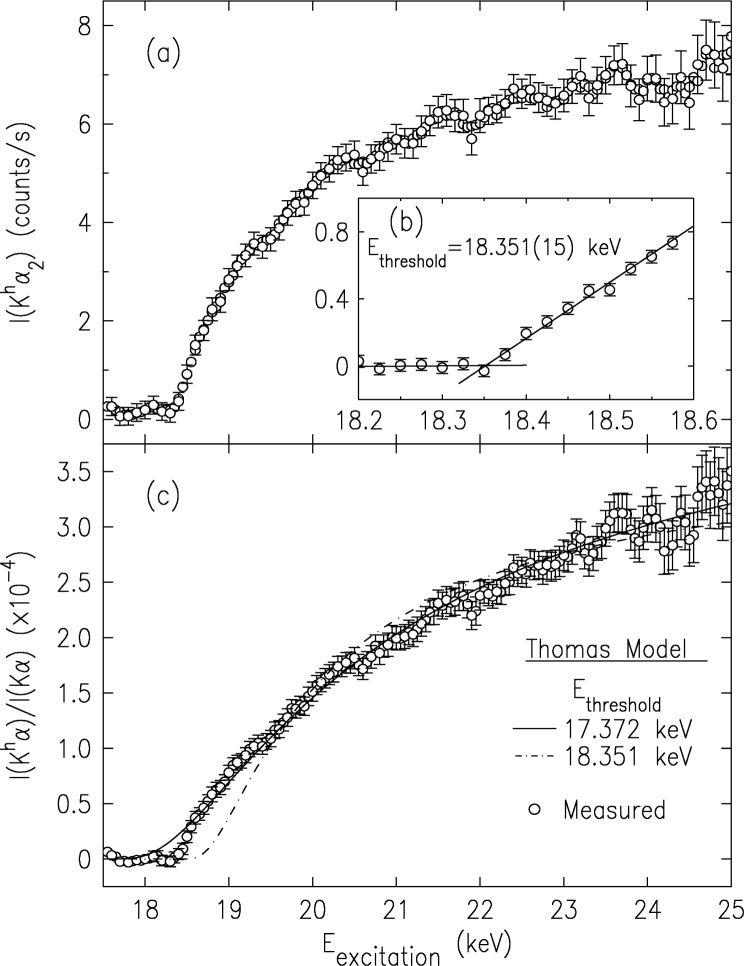
The intensity variation of the CuK*α^h^*_2_ line with incident energy (upper panel). The inset shows a finer measurement of the threshold region, yielding the indicated threshold energy. The lower panel shows the relative intensity variation of the hypersatellite spectrum (points), along fits of the Thomas model discussed in the text (lines). Note that saturation is not reached even at 25 keV, i.e., ≈6.7 keV above threshold.

The evolution of the peak intensity of the K*^h^α*_2_ line with the incident energy, *E*_excitation_, is shown in the upper panel of Fig. 16. While it seems to saturate at ≈ 23 keV, it should be noted that this curve is proportional to the *total* probability of obtaining the initial two-K-hole state in a neutral atom, not to the probability of obtaining the second K hole per directly ionized first K hole created. The last quantity, which is the one most often cited in the literature, has also been evaluated [30], and is shown in the lower panel. A fine scan of the threshold region is shown in the inset of the upper panel, yielding *E*_threshold_ = (18.352 ± 0.015) keV, in excellent agreement with our *Z*+1 approximated [24] $E_{\text{threshold}}^{Z+1}=18.345\text{keV}$ and, to a lesser extent, with our RMCDF-calculated $E_{\text{threshold}}^{\text{DF}}=18.378\text{keV}$. As can be observed, even on this magnified scale the intensity rises from threshold smoothly, as expected of a shake-*off* effect, and no jumps, characteristic of shake-*up* transitions, are discernible. This is in line with shake theory’s prediction of an increasing shake-off/shake-up ratio with increasing *Z* and decreasing principal quantum number, *n*, of the shaken electron’s shell [67,71]. Trends in recent DF calculations for noble gases [71–73] interpolated to the present case of *Z* = 29 and *n* = 1 predict a contribution of less than 1 % from shake-up to the total shake probability. The error bars of the data in Fig. 16 yield an experimental upper limit of <3 % on shake-up contributions to the HS spectrum at threshold in our case.

The intensity of the CuK*α*_3,4_ satellites, discussed above [22], which originate in an *n* = 2 spectator hole transition, were found to saturate at ≈1 keV above threshold, i.e., ≈10 % of the threshold energy. Here, where the spectrum originates in an *n* = 1 spectator hole transition, saturation is not reached even at the highest energy measured, 25 keV. Extrapolation [30] indicates that saturation is reached only around ≈30 keV, making the saturation range here a surprisingly large ≈11 keV or almost 60 % of the threshold energy. These percentage values, when considered with the 3 % saturation range of the *n* = 3 spectator transitions in Ge [56], and the 1 % to 2 % range inferred above (from our near-threshold measurements of the lineshapes of the Cu K*α*_1,2_ and K*β*_1,3_ diagram lines) for the 3*d* spectator hole transitions [57] indicate a very fast increase in the saturation range with decreasing *n* of the spectator hole. We also note that the currently prevailing Thomas model [31,32,74] developed to account for the intensity variation in the adiabatic regime, does not describe well the intensity variation of our measured data in Fig. 16: when the threshold is fixed at the measured value the shape of the fitted curve varies greatly from the measured one, and when the shape is optimized, letting the threshold vary, the refined threshold is ≈1 keV lower than the measured one. A better understanding of the trends in the saturation range's dependence on *n* and *l* of the spectator hole, and the variation of the intensity with incident energy, must await better theoretical calculations in the adiabatic regime.

## 4. Conclusion

The measurements presented above demonstrate the importance of multielectronic transitions in determining the structure of the emission lines of Cu, including the K*α*_1,2_ and K*β*_1,3_ diagram ones. In conjunction with detailed *ab initio* relativistic Dirac-Fock calculations we have shown that such transitions can contribute as much as ≈30 % of the intensity of the diagram lines and are responsible for their well-known asymmetric lineshapes. Inner-shell spectator transitions produce satellite and hypersatellite lines which have been explored as a function of excitation energy using photoexcitation by monochromatized synchrotron radiation. These studies reveal intriguing new effects such as lineshape variations near threshold, unexpectedly long (and, to date, unexplained) saturation ranges strongly dependent upon *n* and *l* of the spectator hole, sensitive dependence of particular features on the coupling scheme and intermediacy, etc. These effects, and, in particular, the near-threshold behaviour, are still far from being understood theoretically. It seems therefore that in spite of an extensive, century-long activity in this field, much still remains to be elucidated. We look forward to new discoveries and the emergence of deeper understandings in this field in the future.

## Figures and Tables

**Table 1:** The peak wavelengths on an absolute scale, *λ*, energies, *E*, full widths at half maximum, *W*, and asymmetry indices, *σ*_w_, of the measured diagram lines. Measurement uncertainties are given in brackets. Wavelength to energy conversion is done using 806 554.477(32) (eV m)^−1^ [75]. Note the 10^−6^ level of uncertainty of the present wavelength results.

Line	*λ* (nm)	*E* (eV)	*W* (eV)	*σ*_w_
CuK*α*_1_	0.154 059 29 (5)	8047.83 (1)	2.29 (2)	1.07
CuK*α*_2_	0.154 442 74 (5)	8027.85 (1)	3.34 (6)	1.36
CuK*β*_1,3_	0.139 223 4 (6)	8905.42 (4)	5.92	

**Table 2:** Positions *E_ij_*, widths *W_ij_*, amplitudes *I_ij_* and integrated intensities *I*_integ_ obtained for the individual Lorentzians from a fit of each measured diagram line by the sum of the minimal number of Lorentzians which provides a reasonable fit to the data. Weighted *R*-factors, *R*_w_, obtained for the fits are also shown.

Line	Comp.	*E_ij_* (eV)	*W_ij_* (eV)	*I_ij_*	*I*_integ_
CuK*α*_1,2_	*α*_11_	8047.837(2)	2.285(3)	0.957(2)	0.579
	*α*_12_	8045.367(22)	3.358(27)	0.090(1)	0.080
	*α*_21_	8027.993(5)	2.666(7)	0.334(1)	0.236
	*α*_22_	8026.504(14)	3.571(23)	0.111(1)	0.105

*R*_w_ (%)			0.7		

CuK*β*_1,3_	*β*_a_	8905.532(2)	3.52(1)	0.757(3)	0.485
	*β*_b_	8903.109(10)	3.52(1)	0.388(2)	0.248
	*β*_c_	8908.462(20)	3.55(3)	0.171(2)	0.110
	*β*_d_	8897.387(50)	8.08(8)	0.068(1)	0.100
	*β*_e_	8911.393(57)	5.31(8)	0.055(2)	0.055

*R*_w_ (%)			1.0		
